# Dr. Cyrus A. Allen

**Published:** 1889-06

**Authors:** 


DR. CYRUS A. ALLEN has removed his dental parlors to the Long building, corner Pearl and Eagle streets.

